# Next-Generation Sequencing of Antibody Display Repertoires

**DOI:** 10.3389/fimmu.2018.00118

**Published:** 2018-02-02

**Authors:** Romain Rouet, Katherine J. L. Jackson, David B. Langley, Daniel Christ

**Affiliations:** ^1^Garvan Institute of Medical Research, Sydney, NSW, Australia; ^2^Faculty of Medicine, St Vincent’s Clinical School, The University of New South Wales, Sydney, NSW, Australia

**Keywords:** antibody display technology, next-generation sequencing, phage display, antibody libraries, *in vitro* selection, antibody therapeutics

## Abstract

*In vitro* selection technology has transformed the development of therapeutic monoclonal antibodies. Using methods such as phage, ribosome, and yeast display, high affinity binders can be selected from diverse repertoires. Here, we review strategies for the next-generation sequencing (NGS) of phage- and other antibody-display libraries, as well as NGS platforms and analysis tools. Moreover, we discuss recent examples relating to the use of NGS to assess library diversity, clonal enrichment, and affinity maturation.

## Introduction

The development of antibody display technology such as phage (1), ribosome (2), yeast (3), and mammalian display (4) has enabled the rapid selection of binders from diverse libraries. These technologies bypass the use of animals and allow for the enrichment of binders within days to weeks. The power of *in vitro* selection technologies relies on a direct physical link between phenotype (displayed antibody construct) and genotype (antibody variable domain genes), allowing for the identification of binders through sequencing of their encoding genes. Multiple rounds of selection are generally required to identify antigen-specific binders, either by binding to a solid support or through cellular sorting (5). In many cases, later rounds of selections tend to be dominated by a handful of clones, which are then further characterized for affinity. Such clonal dominance can reflect genuine selection for high antigen affinity but might also reflect other properties such as superior expression or display. Consequently, clones with superior affinity may be present at low frequency and may not be readily detectable using traditional screening methods such as ELISA (6).

Recent advances in DNA sequencing technologies and computing power over the last decade has led to a dramatic reduction in the cost of sequencing and has simplified data analyses (7). Although initially developed for genomics applications, such as whole-genome sequencing, transcriptome sequencing, and epigenetics, next-generation sequencing (NGS) technology is now increasingly being applied other fields, including to basic and applied immunology. This includes the sequencing of the paired human heavy and light chain repertoire from isolated naïve (8, 9) and antigen-specific B-cells (10, 11), as well as T-cell receptor (12) and antibody display repertoires (13). While most NGS platforms were originally designed for short reads, technology is evolving rapidly, extending both read length and depth. Here, we review recent advances in NGS technology and key applications to phage display and other *in vitro* selection technologies.

### Strategies for NGS of Antibody Repertoires

Traditionally, antibody display libraries are analyzed by isolation of 10^2^–10^3^ clones in combination with Sanger sequencing (5). Although this approach is sufficient to identify dominant clones after selection, or to broadly validate design objectives, the data obtained represent only a limited snapshot of actual library diversity. By contrast, NGS approaches allow for far-greater insights into library diversity by providing up to 10^7^ sequences (approximately 10,000-fold more sequences than Sanger sequencing).

One of the main challenges in the use of NGS for the analysis of antibody selection systems relates to the size of the encoded genes: the smallest antibody fragments (variable domains) range between 300 and 400 bp in length, while the commonly used scFv and Fab antibody fragment formats range from 700 to 800 bp to over 1,500 bp, respectively. While NGS technologies are particularly well suited for high throughput sequencing of short reads (less than 100 bp), many platforms can nevertheless sequence up to 300–400 bp with reasonable throughput. In particular, Illumina Miseq and Hiseq, 454 GS FLX (instrument discontinued), and Ion Torrent PMG are suited for this task (8, 14, 15); in addition, PacBio sequencing generates particularly long reads at the cost of reduced read numbers (Table 1) (16). Long sequences can also be generated by using paired-end reads: this method is particularly useful for scFv formats, enabling the sequencing of multiple CDR regions of V_H_ and V_L_ domains. In addition to analysis of longer antibody fragment sequences, some studies have focused on sequencing the relatively short V_H_ CDR3 repertoire only (23) [which forms the center of the antigen binding site and is a major determinant of antigen binding (24)].

**Table 1:** Next-generation sequencing platforms for the analysis of display libraries.

Platform	Read length	Max. depth	Error type (percentage)	Reference
Illumina Miseq	300 bp PE	40 × 10^6^ reads	Substitutions (~0.1)	(15, 17)
Illumina Hiseq 2500	250 bp PE	600 × 10^6^ reads	Substitutions (~0.1)	(18, 19)
Ion Torrent PMG	400 bp	5.5 × 10^6^ reads	Indels (~1)	(14, 20)
454 GS FLX	Up to 1 kb	1 × 10^6^ reads	Indels (~1)	(13, 21)
PacBio	250 bp–40 kb	0.4 × 10^6^ reads	Indels (~1)	(22)

The use of NGS requires particular attention be paid to sequencing errors (25). DNA amplification inevitably results in polymerase errors, which can be context dependent. Although the error rates of polymerases are generally low (10^−5^–10^−6^ per base), errors will inevitably be present in large NGS datasets that encompass billions of bases. In addition, the NGS technologies themselves can be susceptible to the introduction of errors, such as cluster misamplification and base misincorporation, with frequencies ranging from 10^−2^ (PacBio, Ion Torrent) to 10^−3^ (Illumina). To help identify PCR and sequencing errors, unique molecular identifiers (UMIs; stretches of 8–10 degenerate DNA bases) can be added to primers during the first two cycles of PCR amplification. Reads that share the same UMI have a high probability of being derived from the same original template. Such reads can then grouped after sequencing and used for error correction (26).

### Bioinformatic Tools to Analyze NGS Data

While the analysis of the limited number of clones obtained by Sanger sequencing can be carried out manually, the larger sample size of NGS approaches necessitates the use bioinformatics tools. Following confirmation of the quality of the NGS read data by a tool such as FastQC (27), the data are further processed to clean up reads before analysis of antibody or antibody fragment sequences. The steps undertaken will be highly dependent on the NGS platform utilized and the format of the amplicons but generally will focus on the: removal of adapter sequences [e.g., PRINSEQ (28)], de-multiplexing (if barcodes were used), UMI identification and consensus building, and error correction (26), read quality trimming and filtering [e.g., Trimmomatic (29)] and, if paired-end sequencing was performed, the merging of the read pairs with a program such as PEAR (30). Separate analysis of heavy and light chains may be required for antibody formats such as scFv, where the presence of synthetic linkers can complicate analyses.

Programs such as IMPre (31), IgBLAST (32), IMGT/High V-QUEST (33, 34), and ImmundiveRsity (35), which were originally developed for the analysis of B and T cell receptor repertoires, identify V_H_ and V_L_ germlines as well as V_H_ and V_L_ CDRs. The selection of a tool will depend on the number of NGS reads being analyzed and the computational skill level of the researcher. IgBLAST and IMGT/High V-QUEST are both available as web-based submission systems, with IMGT/High V-QUEST permitting a larger number of reads to be analysis per submission. IMGT/High V-QUEST returns an output format compatible with programs such as Microsoft Excel or OpenOffice, whereas IgBLAST output is text based. The tools use different alignment algorithms, BLAST (IgBLAST) and modified Smith–Waterman (V-QUEST), but both restrict the germline gene repertoires to those defined by the tool’s creators. A stand-alone version of IgBLAST is available, and it has no restriction on the number of input reads, permits the user-defined germline gene databases, provides additional output formats, and can be parallelized on a cluster for processing of large datasets; however, its use does require some command line basics.

Postprocessing of the output of tools such as IgBLAST and IMGT/High V-QUEST is required to generate information about the clone structure within the dataset, and to pair V_H_ and V_L_ domain sequences. Clone structures can be inferred by applying sequence clustering tools, such as CD-hit (36) or UCLUST (37) to CDR3s alone, at either the amino acid or nucleotide sequence level, or to the full-length sequence, to group closely related sequences into “clonal” groups. The choice of parameters will depend on the diversity of the library. Finally, scripts can be used to analyze and summarize the diversity and other compositional characteristics of the library.

A custom pipeline as described earlier requires a level of informatics skills not always available to researchers, therefore, specialized pipelines for the analysis of recombinant antibody libraries, either naïve or *in vitro* selected against particular antigens, have been developed. The AbMining Toolbox is particularly suited for identifying V_H_ CDR3, which is determined by using a hidden Markov model (HMM) that captures the conserved sequences upstream and downstream of the CDR3 (38). N^2^GSAb can rapidly identify germline and V_H_ CDR3 and provides a tool for clustering unique sequences (39). VDJFasta uses an HMM to accurately predict all V_H_ and V_L_ CDRs, as well as the GS linker sequence for scFv fragments, and can generate library diversity plots (13). The ImmuneDB package aligns sequences based on a query sequence, such as a framework region, to delineate CDR regions (40). ImmuneDB also performs mutational and statistical analysis on the sequence library and can construct lineage trees to aid in the interpretation of antigen-selected libraries. More recently, DEAL was developed to better predict library diversity by identifying and correcting sequencing errors (17). In the published example, the library was not generated by PCR but rather by ligation of adapters to avoid any amplification bias and focus on sequencing errors. Reads are clustered using seed sequences of 10–20 bp and analyzed by binary comparison. The clusters are then compared with each read, taking into account the Phred quality score for each base and the error rate of the Phi-X control to identify sequencing errors. A list of software is outlined in Table 2.

**Table 2:** Software for next-generation sequencing analysis.

Software package	Strengths	Reference
IMGT/High V-Quest	Fast germline identification, CDR determination, and batch submission	(34)
ImmunediveRsity	Quality filtering and noise correction	(35)
VDJFasta	Hidden Markov model to determine all CDRs and frequency analysis, very rapid analysis	(13)
N^2^GSAb	Rapid germline and V_H_ CDR3 determination and sequence clustering	(39)
ImmuneDB	Alignment based on sequence query to determine CDRs and frequency	(40)
DEAL	Sequencing error correction before analysis	(17)

It is also possible to outsource the sequencing and/or analysis of antibody libraries to commercial suppliers. Examples include (but are not limited to) CD Genomics and Molecular Cloning Laboratories. Such companies offer a range of options from basic consulting on designing primers for multiplexing and sequencing, to complete analysis from purified DNA or phages.

### Application to Design Validation and the Analyses of Naïve Antibody Libraries

When generating antibody display repertoires, either synthetic or derived from immunized animals, it is important to assess the clonal diversity of the library before selection. Several studies have demonstrated the use of NGS to measure diversity to validate the design of displayed libraries. In an early example, Novimmune designed scFv libraries with both synthetic diversity, using degenerated oligonucleotides, and semi-synthetic diversity, from human or rabbit donors (39). Sequencing of V_H_ CDR3 using the Illumina platform revealed that the synthetic libraries had many more unique clones compared with donor-derived libraries, with between 1–16 and 31–69% clonal redundancy, respectively (39). Intriguingly, the extent of clonal redundancy in the donor-derived libraries suggested an upper limit of human V_H_ diversity of around 2–3 × 10^6^ unique clones. This figure correlates with other NGS studies aimed at determining human B-cell diversity (3–9 × 10^6^) (41). In addition, the Novimmune sequencing results also validated the V_H_ CDR3 length distribution in human antibodies, which closely matched that of the IMGT repertoire (39).

A second example, relates to the Ylanthia synthetic antibody library developed by MorphoSys (21). The library was designed to encode a range of V_H_ CDR3 lengths to closely match the natural human antibody repertoire and was analyzed by the Roche 454 sequencing platform (21). The library was found to be composed of about 95% unique clones, and there was no indication of amplification biases during antibody library construction. In addition, the authors used NGS data to validate V_H_ CDR3 diversity and length, as well as V_H_ and V_L_ germline frequencies.

High throughput sequencing approaches are not limited to human sequences, with a recent study assessing the diversity of rabbit (V_H_ and V_L_) Fab libraries by NGS (Ion PGM) (20). Surprisingly, and unlike human libraries derived from donors, these studies detected very low levels of redundancy within the rabbit libraries, with over 98% of V_H_ clones being of a unique nature (~3 × 10^9^ sequence reads were analyzed).

Next-generation sequencing has also been used to accurately determine library size. A recent study generated a donor-derived V_H_ library for this purpose, which was then sequenced using Illumina adapter ligation (circumventing the need for PCR amplification) (17). Sequencing depth for the V_H_ library exceeded the library size by three-fold suggesting that the diversity was well represented in the NGS output. The authors estimated the minimal functional diversity to be 1.2 × 10^6^ individual unique clones representing just one-fifth of the original number of bacterial clones.

### Application to Affinity Maturation and Epitope Mapping

Next-generation sequencing can also be used to guide selection toward high affinity clones. For example, one seminal study employed NGS to guide maturation of an scFv fragment directed against ErbB2 to a final affinity of 25 pM (resulting in a 158-fold improvement over wild type) (18). Guided by structure-based design, individual CDR regions (excluding V_L_ CDR2) were randomized, selected against ErbB2 antigen, and analyzed by NGS before and after panning. This revealed enrichment of novel sequence motifs at diversified CDR positions, with the exception of V_H_ CDR3, which was enriched toward the wild-type motif (suggesting an already optimal sequence). Next, the most frequent CDR substitutions were combined to generate a secondary library (V_H_ CDR3 being reverted to wild type), which was selected against the target. This resulted in improved affinities of between 300 and 25 pM, compared with the wild-type affinity of 4 nM, highlighting the power of this stepwise approach for affinity maturation.

In a further study, deep mutational scanning analysis using NGS was performed on a humanized version of the anti-EGFR monoclonal cetuximab (42). More specifically, independent V_H_ and V_L_ libraries (encoding over 1,000 single amino acid substitutions at 59 different positions—32 in V_H_ and 27 in V_L_) were selected by mammalian cell display and flow cytometry. Gated populations were analyzed by NGS to identify permissive mutations and to generate a heat map of the antigen binding site. Overall, this strategy identified 67 substitutions that increased affinity, including one mutation with a five-fold *K*_D_ improvement. Similar strategies can also be used to map epitope surfaces, as exemplified by the interaction of *S. aureus* toxin with neutralizing antibodies (43).

## Conclusion

Next-generation sequencing holds great promise for the development of therapeutic monoclonal antibodies, by allowing unprecedented insights into library diversity and clonal enrichment. Although current NGS platforms were not designed with antibody libraries in mind, the technologies are now at a stage where unique sequence insights into all stages of the selection process can be obtained. Moreover, with ongoing advances in sequencing technology, depth and read length is improving continuously: for instance, the PacBio Sequel system generates approximately seven times more sequences than the previous RS II system but maintains its long-read capability (Pacific Biosciences), while nanopore systems such as the MinIOn (Oxford Nanopore) offer the promise of real-time DNA sequencing in combination with ultra-long reads. We conclude that, with NGS technology evolving at a rapid pace, its importance in the sequence analyses of phage- and other antibody-display libraries is likely to continue to increase.

## Author Contributions

RR wrote the manuscript. KJ, DL, and DC edited the manuscript.

## Conflict of Interest Statement

The authors declare that the research was conducted in the absence of any commercial or financial relationships that could be construed as a potential conflict of interest.
